# The effectiveness and safety of eyelid defect reconstruction after sebaceous carcinoma of the eyelid surgery: A protocol for systematic review and meta-analysis

**DOI:** 10.1097/MD.0000000000034531

**Published:** 2023-08-11

**Authors:** Yu Zhao, Rong Bai, Hongyan Hao, Wei Qi, Sheng Li, Jun Li

**Affiliations:** a Department of Ophthalmology, Lanzhou First People’s Hospital, Lanzhou, China.

**Keywords:** effectiveness, eyelid defects, meibomian adenocarcinoma, systematic review

## Abstract

Sebaceous carcinoma of the eyelid is the third most common eyelid malignancy, after basal cell carcinoma and squamous cell carcinoma. It is highly malignant and potentially aggressive. Surgical excision is currently the best treatment option for this condition. Patients often require reconstruction surgery to repair eyelid defects to achieve normal eyelid function and appearance. However, no comprehensive systematic review has assessed the efficacy and safety of eyelid defect reconstruction. This protocol was developed to conduct a systematic review and meta-analysis to evaluate evidence related to the efficacy and safety of reconstruction.

**Methods:**

We will systematically search the Cochrane Library, PubMed, Web of Science, China National Knowledge Infrastructure, WanFang Database, and Chinese Biomedical Literature Database from their inception to February 2023 for studies on eyelid defect reconstruction. We will identify other potential studies using multiple methods such as manual searching. The outcomes were eyelid function, eyelid morphology, patient satisfaction, recurrence rate, metastasis rate, tumor-related mortality, and adverse events. Two researchers will independently screen titles and abstracts, identify full-text studies for inclusion, extract data, and appraise the risk of bias in the included studies. A meta-analysis will be conducted using Review Manager 5.4 and R software. The certainty of evidence will be appraised by grading of recommendations, assessment, development, and evaluation system.

**Results:**

This full-text will adhere to the preferred reporting items for systematic reviews and meta-analyses statement to ensure clarity and completeness of reporting in all phases of the systematic review.

**Discussion:**

This study provides evidence of the efficacy and safety of reconstruction methods for sebaceous carcinoma of the eyelid.

## 1. Introduction

Sebaceous carcinoma of the eyelid (SCE) arises in the glands of Zeis and is associated with hair follicles and meibomian glands.^[1]^ The incidence of SCE varies significantly with race, especially in the Chinese and Japanese populations.^[2]^ Although the incidence varies by region, the high and increasing incidence has made it the third most common eyelid malignancy after basal cell carcinoma and squamous cell carcinoma globally.^[3–5]^ SCE is highly malignant and potentially aggressive.^[1]^ Studies have demonstrated a recurrence rate of 36% after treatment modalities including simple local excision, orbital exenteration, radiation, and chemotherapy, and a metastasis rate of 25% after excision.^[6–12]^ Meanwhile owing to the diversity of clinical and histopathological manifestations of SCE, its diagnosis is challenging.^[13]^ Patients are often misdiagnosed with squamous cell carcinoma or basal cell carcinoma, which often causes treatment delays and worsens the disease, resulting in irreparable damage.^[14,15]^

The current primary treatment for SCE is complete surgical resection followed by eyelid reconstruction.^[2,16]^ The effective excisional surgery involves wide local excision and frozen sectioning or Mohs micrographic surgery.^[17–22]^ Both therapies reduce recurrence, metastasis, and tumor-related mortality by 15.9%, 12.1%, and 6.2%, respectively.^[23]^ Postoperative reconstruction is performed to maintain eyelid function and aesthetics. Multiple materials are used for reconstruction, such as the Hughes flap, switch flap, free tarsus, palatal mucosa, cartilage auriculae, etc.^[24]^ Different surgical techniques are applied in eyelid reconstruction, such as upper blepharoplasty, and Hughes technique.^[25–27]^ Some studies have shown the effectiveness of postoperative reconstruction in improving the appearance of the face, maintaining normal physiological function of the eyelids, and enhancing patient satisfaction.^[24,28–35]^ But findings were inconsistent between studies.

To date, no study has synthesized and compared the reconstruction outcomes of SCE. The efficacy of reconstruction is unclear. Moreover, there is no recommendation concerning SCE reconstruction in the latest clinical practice guidelines.^[15]^ Therefore, a high-quality systematic evaluation program has been used to assess the effectiveness, safety, and availability of the reconstruction.

## 2. Methods

This study protocol was approved by PROSPERO, with registration number CRD42023396392. We follow rigorously the preferred reporting items for systematic reviews and meta-analysis protocol to accomplish the systematic review protocol.^[36]^ Any methodological changes will be reported in the final Systematic Review.

### 2.1. Eligibility criteria

#### 2.1.1. Research types.

Randomized controlled trials, cohort studies, case-control studies, and case studies will be included.^[15]^ Duplicate publications, literature reviews, animal experiments, and irrelevant articles will be excluded.

#### 2.1.2. Participants.

We will include adult patients with a diagnosis of SCE by pathological histological examination.

#### 2.1.3. Interventions/control.

The study includes all repair methods (materials such as lid conjunctival flap, Hughes flap, switch flap, free tarsal joint, labial mucosa, oral mucosa, palatal mucosa, ear cartilage, nasal septum cartilage, and allograft sclera) with or without conventional treatment for SCE, such as chemotherapy and radiotherapy.

#### 2.1.4. Outcome.

Referring to the relevant literature, we focused on eyelid function, eyelid morphology, patient satisfaction, recurrence rate, metastasis rate, tumor-related mortality, and adverse events.^[7,8,23,37–39]^ For eyelid function, any utilized measurement will be included in the outcome, such as the amplitude of the levator excursion from downgaze to upgaze. For eyelid morphology, any utilized measurement to assess lid appearance will be included in the outcome evaluation, such as the width of the palpebral fissure at its widest point and the marginal reflex distance. Patient satisfaction using relevant patient-reported outcome measurement tools will be analyzed.^[39]^

### 2.2. Search strategy

Referring to previous studies and protocol,^[2,40–42]^ we created a comprehensive and detailed search strategy. Specific Boolean search strings were constructed. Six electronic databases will be retrieved from their establishment until February, 2023: PubMed, Cochrane Library, Web of Science, Chinese Biomedical Literature Database, China National Knowledge Infrastructure, and Wan Fang Database. Furthermore, we will identify other potentially eligible studies by tracking references included in studies and previous systematic reviews, checking gray literature, and performing manual searches. We have no restrictions on the language or publication status. The search strategy is shown in Table 1, using PubMed retrieval as an example. Additional database search strategies are presented in Appendix 1, http://links.lww.com/MD/J404 (Supplemental Content, which demonstrates all the search strategies).

**Table 1:** Retrieval strategy of PubMed.

Number	Search term
#1	“meibomian gland carcinoma”[Title/Abstract] OR “meibomian gland carcinomas”[Title/Abstract] OR “meibomian adenocarcinoma”[Title/Abstract] OR “meibomian adenocarcinomas”[Title/Abstract] OR “meibomian carcinoma”[Title/Abstract] OR “meibomian carcinomas”[Title/Abstract] OR “sebaceous gland carcinoma”[Title/Abstract] OR “sebaceous gland carcinomas”[Title/Abstract] OR “sebaceous adenocarcinoma”[Title/Abstract] OR “sebaceous adenocarcinomas”[Title/Abstract] OR “sebaceous carcinoma”[Title/Abstract] OR “sebaceous carcinomas”[Title/Abstract] OR “carcinoma of meibomian gland”[Title/Abstract] OR “carcinoma of sebaceous gland”[Title/Abstract] OR “meibomian gland tumor”[Title/Abstract] OR “meibomian gland tumors”[Title/Abstract]
#2	repair*[Title/Abstract] OR mend*[Title/Abstract] OR revamp*[Title/Abstract] OR renovat*[Title/Abstract] OR restor*[Title/Abstract] OR reconstruct*[Title/Abstract] OR rebuild*[Title/Abstract] OR reestablish*[Title/Abstract] OR rehabilitat*[Title/Abstract]
#3	#1 AND #2

### 2.3. Study screening

**Figure 1.**
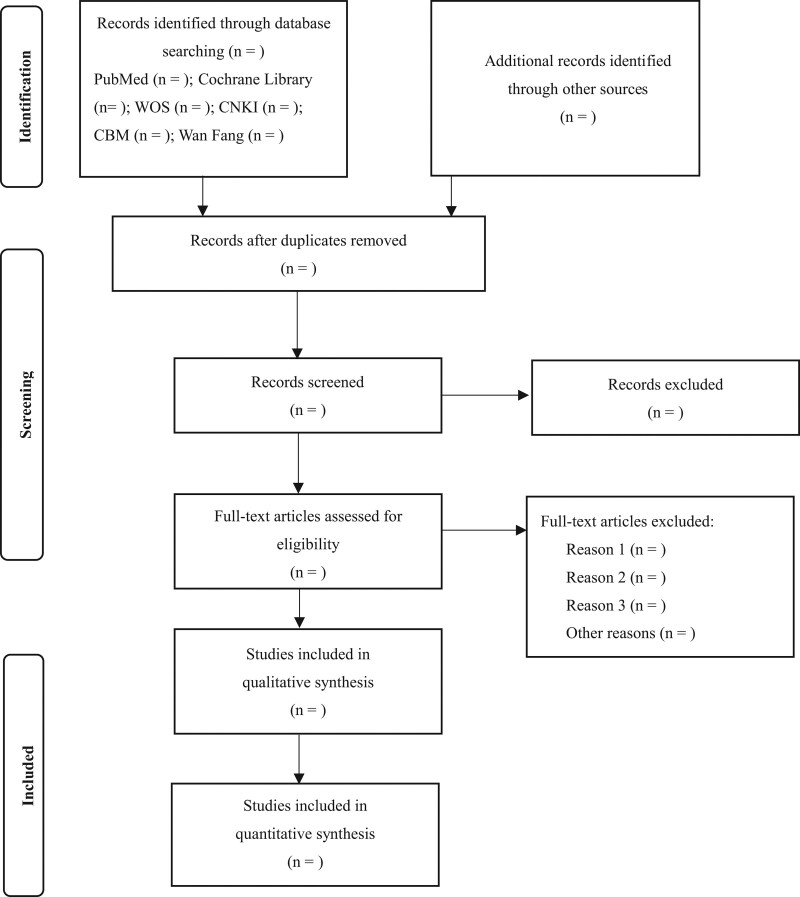
Preferred reporting items for systematic reviews and meta-analysis (PRISMA) flow chart for the study selection process. We will use the preferred reporting items for systematic reviews and meta-analyses (PRISMA) flow chart to record the study selection process. The arrows in the flow chart represent work in progress. “n” represents the number of studies. **Identification:** Researchers will search PubMed, Cochrane Library, Web of Science (WOS), Chinese Biomedical Literature Database (CBM), China National Knowledge Infrastructure (CNKI), Wan Fang Database. **Identification:** Researchers will identify other potentially eligible studies by other method. **Screening**: Rigorous study-selection process including “duplicate records screening,” “title and abstract screening,” and “full text screening.” **Screening:** “Records screened” section will record the number of exclusions. **Screening**: “Full-text articles assessed for eligibility” section will document the list of excluded studies and the reasons for studies exclusion. **Included:** Determining the number of studies to include in the quantitative and qualitative analyses.

We will follow a rigorous study-selection process. Two reviewers (YZ and WQ) will be prescreened, and formal screening will not be performed until both reviewers have achieved at least 85% consistency in screening. Subsequently, the 2 reviewers will independently browse and select potentially eligible studies through titles and abstracts using Rayyan (https://www.rayyan.ai/). Furthermore, we will evaluate the full text to identify eligible studies using the synthesis form. We will first randomly select 5 studies, and 2 reviewers (YZ and WQ) will independently conduct full-text pilot screening. The authors will then discuss and compare the consistency of the screening results. We will conduct a formal full-text screening followed by this study. Any conflicts and disagreements will be resolved by a third reviewer (JL) through discussion and consultation. A preferred reporting items for systematic reviews and meta-analysis flow chart will be used to record the study selection process (see Fig. 1).

### 2.4. Extraction and management of the data

Data extracted from the studies will include basic characteristics (article ID, article title, author name, publication year, study design, country(ies) where study is based, sample size, diagnosis, diagnostic criteria, presenting symptoms, tumor location, tumor size, tumor stage, tumor margin status, treatment, adjuvant treatment, follow-up time), intervention and control (intervention name, intervention description, control name, control description), outcome (eyelid function, eyelid morphology, patient satisfaction, recurrence rate, metastasis rate, tumor-related mortality, adverse events), and study quality assessment. Two reviewers (WQ and RB) will use a standardized form and Microsoft Excel (Microsoft Corporation, Washington, U.S.) spreadsheets to independently extract data from each eligible study. Any adjustments and judgments will be resolved by a third reviewer (JL).

### 2.5. Risk of bias assessment

Two reviewers (HH and SL) will independently assess the methodological quality of the study using different risk of bias assessment tools based on the study design. Any conflicts and disagreements will be resolved by a third reviewer (SL). First, we will randomly assess 5 studies to resolve disagreements and achieve a consensus before a formal assessment.

Randomized controlled studies will be evaluated using the Cochrane risk-of-bias tool (RoB 2.0) for individually randomized parallel-group and crossover trials.^[43]^ The following domains will be assessed: bias arising from the randomization process, bias due to deviations from intended interventions, bias due to missing outcome data, bias in the measurement of the outcome, and bias in the selection of the reported result. Excel will be used to input answers given to signaling questions. Each domain judgement will be categorized as “low risk of bias,” “some concerns,” or “high risk of bias.” The overall judgments will be categorized as follows: low risk of bias(the study is judged to be at low risk of bias for all domains for this result), some concerns(the study is judged to raise some concerns in at least one domain for this result, but not to be at high risk of bias for any domain), and high risk of bias(the study is judged to be at high risk of bias in at least one domain for this result or the study is judged to have some concerns for multiple domains in a way that substantially lowers confidence in the result).

Non-randomized studies will be evaluated using the risk of bias in nonrandomized studies of interventions (ROBINS-I) tool.^[44]^ The following domains will be assessed: bias due to confounding factors, bias in the selection of participants into the study, bias in the classification of interventions, bias due to deviations from intended interventions, bias due to missing data, bias in the measurement of outcomes, and bias in the selection of the reported results. Each domain judgement will be categorized as “low risk,” “moderate risk,” “serious risk,” and “critical risk.” The overall risk of bias judgement will be categorized as follows: low risk of bias (the study is judged to be at low risk of bias for all domains), moderate risk of bias (the study is judged to be at low or moderate risk of bias for all domains), serious risk of bias (the study is judged to be at serious risk of bias in at least one domain, but not at critical risk of bias in any domain), critical risk of bias (the study is judged to be at critical risk of bias in at least one domain), and no information (there is no clear indication that the study is at serious or critical risk of bias, and there is a lack of information in 1 or more key domains of bias).

### 2.6. Statistical analysis

For controlled studies, we will calculate risk ratios or mean difference and the corresponding 95% credible intervals between the intervention and control groups. If the outcomes used different scales, we will use standardized mean differences. Heterogeneity among individual studies will be assessed using forest plots and quantified using I^2^ statistics.^[45]^ For case studies, the combined composition ratios and their 95% confidence intervals will be calculated using the meta-analysis approach. Considering potential heterogeneity, a random-effects model will be used to combine the data to obtain more conservative results.^[46]^ We consider a *P* value <.05 (*P* < .05) to be statistically significant. If >10 trials report the same results, funnel plots will be utilized to assess potential publication bias.^[47,48]^

If applicable, we will perform several subgroup analyses to test interactions according to repair methods, timing of treatment, and baseline mean age, and conduct meta-regression analyses based on the length of follow-up, year of publication, sex, and race. Sensitivity analyses may be conducted by high or unknown risk of bias study, high risk or unknown risk of bias of the different domains, study of a follow-up of less than half a year and fixed effect model.

Statistical analyses will be performed using RevMan (version 5.4; The Cochrane Collaboration), the *meta* package in R (version 4.0.3; R Project for Statistical Computing; R Core Team, Vienna, Austria), and JAGS version 4.3.0 (Just Another Gibbs Sampler).

### 2.7. Certainty of evidence assessment

Two reviewers (YZ and WQ) will assess the certainty of evidence using the grading of recommendations, assessment, development, and evaluation approach.^[49,50]^ Any disagreement will be resolved by a third author (SL). The grading of recommendations, assessment, development, and evaluation approach considers 5 downgraded factors and 3 upgraded factors to rate the certainty of evidence to very low, low, moderate, and high certainty. The 5 downgraded factors are “Limitations to study quality (risk of bias),” “Inconsistency,” “Indirectness of evidence,” “Imprecision,” and “Probability of publication bias.” The 3 upgraded factors are “Large magnitude of effect,” “Dose-response gradient,” and “Residual confounding would further support inferences regarding the treatment effect.”

### 2.8. Ethics and dissemination

Given that no patients will be recruited and no data gathered from patients, ethical approval is not required to conduct this review. The results of this study will be disseminated in a peer-reviewed journal.

## 3. Discussion

SCE is the third most common malignant eyelid tumor.^[23]^ Despite the low average incidence, its high malignancy and aggressiveness often make presents patients with pain and severely decreases their quality of life, even resulting in death.^[51]^ Surgery is the preferred treatment option, and its value in improving the recurrence, mortality, and metastasis rates is significant.^[23]^ The surgically damaged area must be reconstructed to maintain normal physiological function and aesthetics of the eyelid. A multiplicity of reconstructive methods is available. However, no studies have systematically summarized the efficacy of SCE reconstructive surgery, making the efficacy of various reconstruction methods unclear. This rigorous systematic review will provide the best evidence to support the optimal recommendations for the clinical treatment of reconstruction methods. However, this study may have potential limitation. We will include studies with different reconstruction types, which may lead to high heterogeneity, and we will consider addressing this issue through randomized models and statistical analysis.

## Acknowledgments

We would like to thank Editage (www.editage.cn) for English language editing.

## Author contributions

**Conceptualization:** Jun Li.

**Investigation:** Yu Zhao, Rong Bai, Wei Qi.

**Methodology:** Yu Zhao, Rong Bai, Hongyan Hao, Wei Qi, Jun Li.

**Project administration:** Yu Zhao, Jun Li.

**Supervision:** Sheng Li, Jun Li.

**Writing – original draft:** Yu Zhao.

**Writing – review & editing:** Yu Zhao, Jun Li.

## Supplementary Material


