# Supplementary material for: The effectiveness and safety of eyelid defect reconstruction after sebaceous carcinoma of the eyelid surgery: A protocol for systematic review and meta-analysis
Source: Medicine (Baltimore). 2023 Aug 11;102(32):e34531. doi: 10.1097/MD.0000000000034531 (PMC10419430; doi:10.1097/MD.0000000000034531)
Supplement: Supplementary file 1 [file medi-102-e34531-s001.pdf]

The effectiveness and safety of eyelid defect reconstruction after sebaceous carcinoma of the eyelid surgery: a protocol for systematic review and meta-analysis

Running Title: Reconstruction for eyelid defect

Yu Zhao, Wei Qia, Rong Bai, Hongyan Hao, Sheng Li, Jun Li

CNKI

Journal Theses and Dissertations

Search Strategy:

#	Searches	Results
1	SU = ('睑板腺癌' + '麦氏腺癌' + '迈博姆氏腺癌' + '睑板腺肿瘤')	Not yet searched
2	TKA = ('睑板腺癌' + '麦氏腺癌' + '迈博姆氏腺癌' + '睑板腺肿瘤')	Not yet searched
3	1 OR 2	Not yet searched
4	SU = ('修复' + '修补' + '再造' + '重建')	Not yet searched
5	TKA = ('修复' + '修补' + '再造' + '重建')	Not yet searched
6	4 OR 5	Not yet searched
7	3 AND 6	Not yet searched

WANFANG

Journal Theses and Dissertations

Search Strategy:

#	Searches	Results
1	主题:("睑板腺癌" or "麦氏腺癌" or "迈博姆氏腺癌" or "睑板腺肿瘤")	Not yet searched
2	主题:("修复" or "修补" or "再造" or "重建")	Not yet searched
3	1 AND 2	Not yet searched

CBM

Search Strategy:

#	Searches	Results
1	"睑板腺癌"[常用字段:智能] OR "麦氏腺癌"[常用字段:智能] OR "迈博姆氏腺癌"[常用字段:智能] OR "睑板腺肿瘤"[常用字段:智能]	Not yet searched
2	"修复"[常用字段:智能] OR "修补"[常用字段:智能] OR "再造"[常用字段:智能] OR "重建"[常用字段:智能]	Not yet searched
3	1 AND 2	Not yet searched

PubMed

Search Strategy:

#	Searches	Results

1	"meibomian gland carcinoma"[Title/Abstract] OR "meibomian gland carcinomas"[Title/Abstract] OR "meibomian adenocarcinoma"[Title/Abstract] OR "meibomian adenocarcinomas"[Title/Abstract] OR "meibomian carcinoma"[Title/Abstract] OR "meibomian carcinomas"[Title/Abstract] OR "sebaceous gland carcinoma"[Title/Abstract] OR "sebaceous gland carcinomas"[Title/Abstract] OR "sebaceous adenocarcinoma"[Title/Abstract] OR "sebaceous adenocarcinomas"[Title/Abstract] OR "sebaceous carcinoma"[Title/Abstract] OR "sebaceous carcinomas"[Title/Abstract] OR "carcinoma of meibomian gland"[Title/Abstract] OR "carcinoma of sebaceous gland"[Title/Abstract] OR "meibomian gland tumor"[Title/Abstract] OR "meibomian gland tumors"[Title/Abstract]	Not yet searched
2	repair*[Title/Abstract] OR mend*[Title/Abstract] OR revamp*[Title/Abstract] OR renovat*[Title/Abstract] OR restor*[Title/Abstract] OR reconstruct*[Title/Abstract] OR rebuild*[Title/Abstract] OR reestablish*[Title/Abstract] OR rehabilitat*[Title/Abstract]	Not yet searched
3	1 AND 2	Not yet searched

Web of Science

Science Citation Index Expanded (SCI-EXPANDED)

Social Sciences Citation Index (SSCI)

Search Strategy:

#	Searches	Results
1	TS= ("meibomian gland carcinoma" OR "meibomian gland carcinomas" OR "meibomian adenocarcinoma" OR "meibomian adenocarcinomas" OR "meibomian carcinoma" OR "meibomian carcinomas" OR "sebaceous gland carcinoma" OR "sebaceous gland carcinomas" OR "sebaceous adenocarcinoma" OR "sebaceous adenocarcinomas" OR "sebaceous carcinoma" OR "sebaceous carcinomas" OR "carcinoma of meibomian gland" OR "carcinoma of sebaceous gland" OR "meibomian gland tumor" OR "meibomian gland tumors")	Not yet searched
2	TS= (repair* OR mend* OR revamp* OR renovat* OR restor* OR reconstruct* OR rebuild* OR reestablish* OR rehabilitat*)	Not yet searched
3	1 AND 2	Not yet searched

Cochrane Library

Search Strategy:

#	Searches	Results
1	("meibomian gland carcinoma"):ti,ab,kw OR ("meibomian gland carcinomas"):ti,ab,kw OR ("meibomian adenocarcinoma"):ti,ab,kw OR ("meibomian adenocarcinomas"):ti,ab,kw OR ("meibomian carcinoma"):ti,ab,kw OR ("meibomian carcinomas"):ti,ab,kw OR ("sebaceous gland carcinoma"):ti,ab,kw OR ("sebaceous gland carcinomas"):ti,ab,kw OR ("sebaceous adenocarcinoma"):ti,ab,kw OR ("sebaceous adenocarcinomas"):ti,ab,kw OR ("sebaceous	Not yet searched

	carcinoma"):ti,ab,kw OR ("sebaceous carcinomas"):ti,ab,kw OR ("carcinoma of meibomian gland"):ti,ab,kw OR ("carcinoma of sebaceous gland"):ti,ab,kw OR ("meibomian gland tumor"):ti,ab,kw OR ("meibomian gland tumors"):ti,ab,kw	
2	(repair*):ti,ab,kw OR (mend*):ti,ab,kw OR (revamp*):ti,ab,kw OR (renovat*):ti,ab,kw OR (restor*):ti,ab,kw OR (reconstruct*):ti,ab,kw OR (rebuild*):ti,ab,kw OR (reestablish*):ti,ab,kw OR (rehabilitat*):ti,ab,kw	Not yet searched
3	1 AND 2	Not yet searched
